# Key Parameters to Promote Granularization of Lath-Like Bainite/Martensite in FeNiC Alloys during Isothermal Holding

**DOI:** 10.3390/ma11101808

**Published:** 2018-09-24

**Authors:** Meriem Ben Haj Slama, Nathalie Gey, Lionel Germain, Kangying Zhu, Sébastien Allain

**Affiliations:** 1 Institut Jean Lamour UMR Université de Lorraine, CNRS 7198 Nancy, France; sebastien.allain@univ-lorraine.fr; 2 Laboratoire d’Etude des Microstructures et de Mécanique des Matériaux (LEM3), UMR, Université de Lorraine, F-57000 Metz, France; nathalie.gey@univ-lorraine.fr (N.G.); lionel.germain@univ-lorraine.fr (L.G.); 3 Laboratory of Excellence on Design of Alloy Metals for low-mAss Structures (DAMAS), Université de Lorraine, F-57000 Metz, France; 4 ArcelorMittal Maizières Research SA, Automotive Product Center, Voie Romaine, BP30320, 57283 Maizières-les-Metz CEDEX, France; kangying.zhu@arcelormittal.com

**Keywords:** steel, bainite, martensite, isothermal treatment, mechanical properties, austenite reconstruction, variant

## Abstract

The stability of lath-like microstructures during low-temperature isothermal ageing was analyzed in a Fe5Ni0.33C (in wt %) steel. The microstructures were characterized using Scanning Electron Microscopy (SEM) coupled with Electron Backscatter Diffraction (EBSD). Advanced orientation data processing was applied to quantify the hierarchical and multiscale organization of crystallographic variants subdividing Prior Austenite Grains (PAG) into packets/blocks/sub-blocks. The result shows that ferrite laths of martensite or lower bainite are stable, whatever the ageing temperature (up to 380 °C). On the contrary, a granularization process is triggered when microstructures contain a fraction of upper bainite. This metallurgical evolution corresponds to a rapid and significant change of the ferrite matrix involving a disappearance of 60° disoriented blocks. The phenomenon affects in turn the mechanical properties. The final microstructures obtained after isothermal holding look like granular bainite, which raises some questions about the classification of bainite.

## 1. Introduction

Similar to martensite, bainite is a phase transformation product of austenite appearing at low temperature in conventional carbon steels. In most cases, its microstructure can be described as a non-polygonal ferrite matrix containing or not second phases. Depending on the chemical composition of the studied steel and the thermomechanical treatment, a wide range of bainite microstructures can be observed at the micrometer scale. That explains why numerous classifications were proposed in the past [1,2,3,4,5]. The first classifications were derived from Optical Microscopy (OM) or Scanning Electron Microscopy (SEM) observations and thus, mainly based on the apparent morphology and spatial distribution of the constituting phases after etching.

From the late 1990s, the progress in orientation-based microscopy [6,7,8] have permitted investigating in a statistical way and with a high accuracy the crystallographic disorientations between features constituting bainite. As bainite exhibits an orientation relationship (OR) with its austenite parent phase [9,10,11,12] due to its associated shear transformation strain, the analysis of disorientations between ferrite variants has been the ground for new crystallography-based classifications [13,14,15]. They have permitted to rationalize the mechanical properties of lath-like bainite, depending on their transformation temperatures. Thus, crystal orientation-based techniques have become essential to study bainite steels.

These later classifications focus on the crystallographic variant arrangement within PAGs (Prior Austenite Grains). This arrangement is inherited from the mechanism of the transformation with respect of an OR between the austenite and ferrite phases, similar to martensite steels [5,9,10,11,12]. The crystallographic variants are grouped in packets (neighboring variants sharing the same habit plane), further divided into highly disoriented blocks (>45°, mainly 60°) themselves divided in low disoriented sub-blocks (<20°) (or variants) [16]. The different ORs have been reported depending on the chemical composition of the steel and the manufacturing conditions, but the multiscale and hierarchical organization of the structure discussed above has unanimous support.

Thanks to their good balance between ductility and strength, bainite/martensite steels are products widely used in industry [17]. Their strength in particular results from their refined microstructure (block/lath sizes), the dense carbide distribution (if any), the carbon in solid solution and the high density of dislocations inherited from the shear transformations at low temperature [18,19,20]. However, all these microstructure features are prone to evolve during further thermal treatments. In the case of martensite steels, these phenomena are known as tempering processes and have been widely studied in the past [21,22,23]. This versatility offers a fantastic lever to metallurgists to tune the final properties of steels. On the contrary, very few published studies focus on these processes in bainite steels; the ferrite matrix is thought to be stable during further thermal treatments, and only the lath structure is thought to thicken to some extent [24]. Significant evolution of lath-bainite microstructure was reported only after very long isothermal holding at 500 °C for ~1 year, where mechanisms such as carbide coarsening and ferrite recrystallization were observed [20]. Only severe thermal treatment seems able to induce significant evolution of bainite microstructures, contrary to martensite steels and despite their numerous similarities.

To rationalize the comparison of different isothermal treatments at different temperatures, Hollomon and Jaffe [25] have defined a tempering parameter H_HJ_ to describe time/temperature equivalence such as:

H_HJ_ = T (C + log(t)) (1)

where C is a dimensionless constant proposed by the authors to be ~20 for steels with carbon contents of 0.25–0.4 wt %. T is the temperature in kilokelvin and t is the time in hour. The severity of the thermal treatment increases with H_HJ_. The authors reported that the mechanical strength of bainite steels dropped with further tempering over a threshold H_HJ_ value of 18, whatever the bainite microstructure [25]. This value corresponds for instance to a heat treatment of 80 days at 500 °C. The same behavior was confirmed by different authors for alloyed steels (Fe-0.5C-0.8Mn-0.2Si-0.9Ni-1.1Cr-0.5Mo and Fe-0.42C-0.7Mn-0.5Si-0.8Ni-2.5Cr-1Mo in wt % [26], and Fe-0.20C-0.3Si-1.4Mn-0.7Ni and Fe-0.13C-0.2Si-0.6Mn-2.4Cr-0.9Mo in wt % [27]). The main mechanism of softening was carbide coarsening and in some cases a stress-release process.

However, our recent work on a model alloy Fe-5Ni-0.13C (wt %) reported a softening of bainite for an unexpected low value of H_HJ_ (12.8) [28]. The recorded drop in hardness and yield strength was 45 HV and 160 MPa, respectively. According to this result, microstructural evolution of bainite can be largely faster than what was reported in the previous literature, and could occur at lower temperatures (Ms + 20 K; Ms is the martensite start temperature of the alloy). In fact, a fast granularization of lath-like bainite was observed during the studied isothermal holding.

The present work aimed at a better understanding of this lath granularization process in a similar Ni-based steel (Fe-5Ni-0.33C in wt %). Different microstructures (lower or upper bainite, martensite and mixed microstructures) and different ageing time/temperatures were considered. They were characterized by high resolution EBSD analysis coupled with crystallographic reconstruction of PAGs using software developed by our group.

The result of this research is of major importance for various industrial applications (pipelines, nuclear reactor vessels, car-to-ground connecting components, engine components, etc.); it helps to better understand and predict the evolution of the properties of bainite/martensite steels during manufacturing and in service conditions.

## 2. Materials and Methods

### 2.1. Material

The composition of the investigated steel was Fe-0.33C-5Ni (wt %). It is a model alloy compared to industrial steels. In particular, Mn has been substituted with Ni to avoid composition heterogeneities and segregations. A 15 kg ingot was cast in a vacuum induction furnace. After a 2 h homogenization treatment at 1200 °C, it was hot and then cold rolled, respectively, to the thicknesses of 3 mm and 1 mm. Dilatometric samples of 10 mm × 3 mm were cut from this as-rolled plate for further controlled heat treatments. Vickers Hardness on Zwick-Roell/Indentec machine (Zwick France, Metz, France), as well as SEM/EBSD analysis were performed on the treated samples. All microstructure analyses were performed at quarter thickness of the sample to avoid possible decarburized layers at the surface.

### 2.2. Controlled Heat Treatments

Three families of microstructures were obtained by controlled heat treatment performed in a laboratory-made rapid-cycle dilatometer (RCD) (Institut Jean Lamour, Nancy, France): (1) high temperature bainite; (2) low temperature bainite; and (3) martensite-based microstructures (either fully martensite or mixed bainite/martensite). For each family of microstructures, two metallurgical states were generated: (i) the so-called “transformed” state (T) was obtained by quenching the samples directly after the bainite transformation was completed at the considered temperature (according to the dilatometric signal), or directly from the austenite domain to obtain fully martensite microstructure; and (ii) the so-called “Aged” state (A) was obtained by further holding (either at the transformation temperature or at higher/lower temperatures). This “aged” state corresponds in fact to a tempering in the case of a martensite-based microstructure.

**Figure 1:**
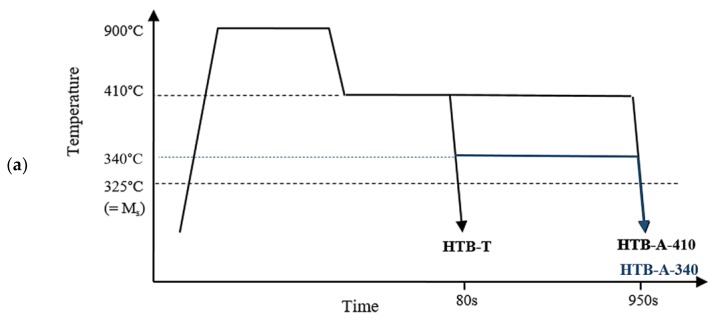

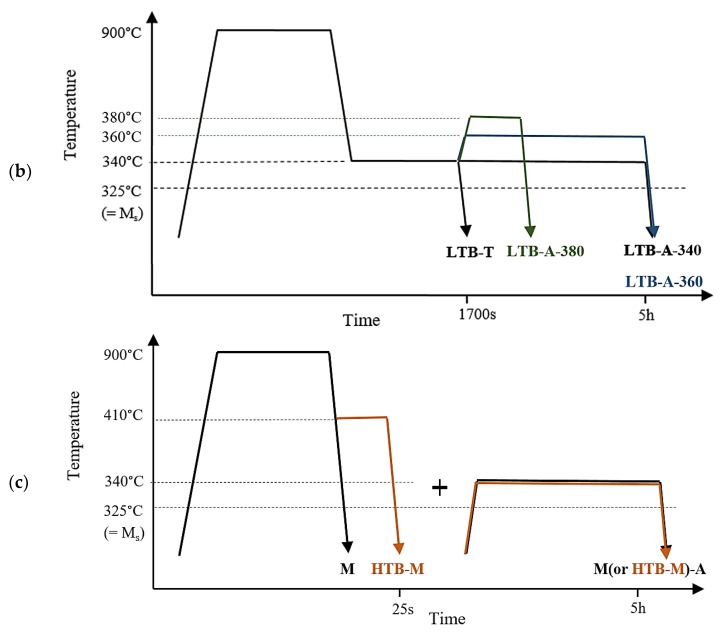
Heat treatments to obtain: (**a**) High Temperature Bainite HTB-type microstructures; (**b**) Low Temperature Bainite LTB-type microstructures; and (**c**) martensite-based microstructures (T stands for as-transformed and A for as-aged conditions).

The different heat treatments associated to each type of microstructures are further described in the coming subsections and summarized in Figure 1 and Table 1. This table also indicates Vickers hardness results (ten measurements were averaged for each reported values) and H_HJ_ parameters calculated according to Equation (1).

Prior to bainite and martensite transformations, all samples were first austenitized for 5 min at 900 °C (1173 K) and rapidly cooled at 70 °C/s to:

- the transformation temperature in the case of bainite containing microstructures; and
- room temperature for fully martensite microstructures.

The austenite holding time was optimized to ensure a homogenous austenite composition with a conventional average grain size of 17 µm. Moreover, the cooling was fast enough to avoid the ferrite transformation, as confirmed by the dilatometry measurements and the microstructure investigations.

#### 2.2.1. High Temperature Bainite (HTB)

HTB was isothermally formed at 410 °C (683 K), i.e., Ms + 85 °C. At this temperature, the transformation was completed after 80 s. The sample in the “transformed” state (HTB-T) was directly quenched after the transformation was finished. Two additional samples were further aged without any intermediate quench (In contrast to standard definition, the “aged” condition is here obtained without intermediate cooling): sample HTB-A-410 was maintained at the transformation temperature for 870 s and sample HTB-A-340 at 340 °C (613 K) for 870 s (see Figure 1a and Table 1a).

#### 2.2.2. Low Temperature Bainite

LTB was isothermally formed at 340 °C, i.e., Ms + 15 °C. At this temperature, the transformation was completed after 1700 s. The sample in the “transformed” state (LTB-T) was directly quenched after the transformation was finished. Three additional samples were further aged at different temperatures, without any intermediate quench: sample LTB-A-340 was maintained at the transformation temperature for 5 h, sample LTB-A-360 at 360 °C for 5 h and sample LTB-A-380 at 380 °C for 1800 s (see Figure 1b and Table 1b).

#### 2.2.3. Martensite-Based Microstructures

A fully martensite structure (sample M) was also obtained by fast quenching from the austenite domain. Finally, a mixed bainite–martensite microstructure (sample HTB-M) was formed by an isothermal holding at 410 °C for 25 s followed by a quench. In the latter case, the resulting microstructure was supposed to contain 60% of HTB and 40% of fresh martensite.

For each type of microstructure, an additional sample was further tempered at 340 °C, i.e., Ms + 15 °C. They are referred to as M-A and HTB-M-A samples (Figure 1c and Table 1c).

### 2.3. Microstructure and Microtexture Characterization

The microstructures were characterized by electron microscopy with the JEOL 6500F Field Emission Gun-SEM (JEOL, Peabody, MA, USA) equipped with the Oxford-instrument EBSD system (Nordlys II CCD camera (Oxford-instrument, Gometz-la-Ville, France) and AZTec acquisition software) (AZTecHKL, Oxford-instrument, Gometz-la-Ville, France ). For the EBSD measurements, the samples were mechanically polished down to 1-µm diamond paste and finished with colloidal silica. Orientation maps were acquired at an acceleration voltage of 15 kV, with a current of 3.3 nA, allowing a signal of acceptable quality for a camera resolution of 336 × 256 pixels (i.e., with a 4 × 4 binning) and with an integration time of 48 ms per shot without averaging. Under these conditions, it is estimated that the spatial resolution of the diffraction signal is about 50/70 nm for steels (http://lionelgermain.free.fr/merengue2.htm) [29,30]. For a 1500 × 1150 pixel field with a 0.1 μm step size, the acquisition time is approximately of 24 h.

The disorientation maps were plotted from EBSD data as well as the pixel-to-pixel disorientation histogram. The maps highlight low disorientations (<20°) corresponding to sub-block boundaries, high disorientations (>45°, mainly 60°) corresponding to block and packet boundaries and random disorientations (between 20° and 45°) corresponding to PAG boundaries. Notice that only the disorientation angles were considered to characterize the boundaries of the bainite/martensite microstructures, leaving out disorientation axes.

After EBSD studies, some samples were etched with Picral solution to reveal carbides and characterized by SEM using an in-lens detector.

**Figure 2:**
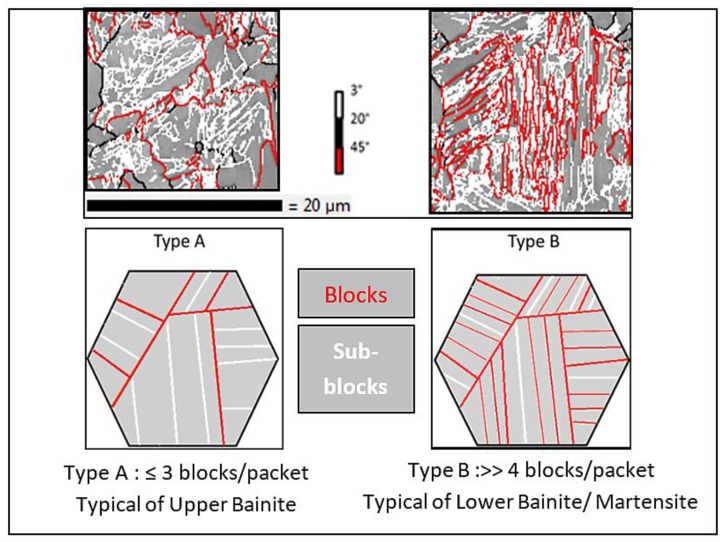
Crystallographic variant organizations of Type A and Type B microtextures (from the top to the bottom: experimental disorientation maps of a single PAG and corresponding schematic organization of blocks and packets inside a PAG)—PAG, packet/block, and sub-block boundaries are plotted in black, red and white respectively.

MERENGUE2 in-house software was applied to the ESBD maps to identify the prior austenite grains [30]. The PAG size was about 17 µm after five minutes at 900 °C. DECRYPT (Direct Evaluation of CRYstallographic Phase Transformation) in-house software was then applied to further analyze the variant arrangement in packets and blocks inside the PAGs and quantify the number of blocks and packets per PAG. Using this approach, two crystallographic types of variant arrangements inside PAGs were identified on the fully transformed microstructures (bainite or martensite) and called Type A and Type B microstructures. Figure 2 shows the typical disorientation map obtained on two PAGs mainly transformed into Type A and Type B microstructures. For the sake of readability, a simplified schematic illustration is also provided. Type A is characterized by a small number of blocks per packet. This number is statistically found to be less than three in the majority of the studied experimental configurations. On the contrary, Type B microstructure is characterized by a higher number of blocks per packet, largely more than four statistically (21 on average). Type A is thus representative of upper bainite microtexture while Type B is representative of lower bainite or martensite microtexture. This crystallographic classification based on the number of blocks per packet is in agreement with recent bainite classifications; in particular the ones suggested by Furuhara et al. [14] and Takayama et al. [15].

## 3. Results

### 3.1. Evolution of Bainite Microstructures during Extended Isothermal Holding

**Figure 3:**
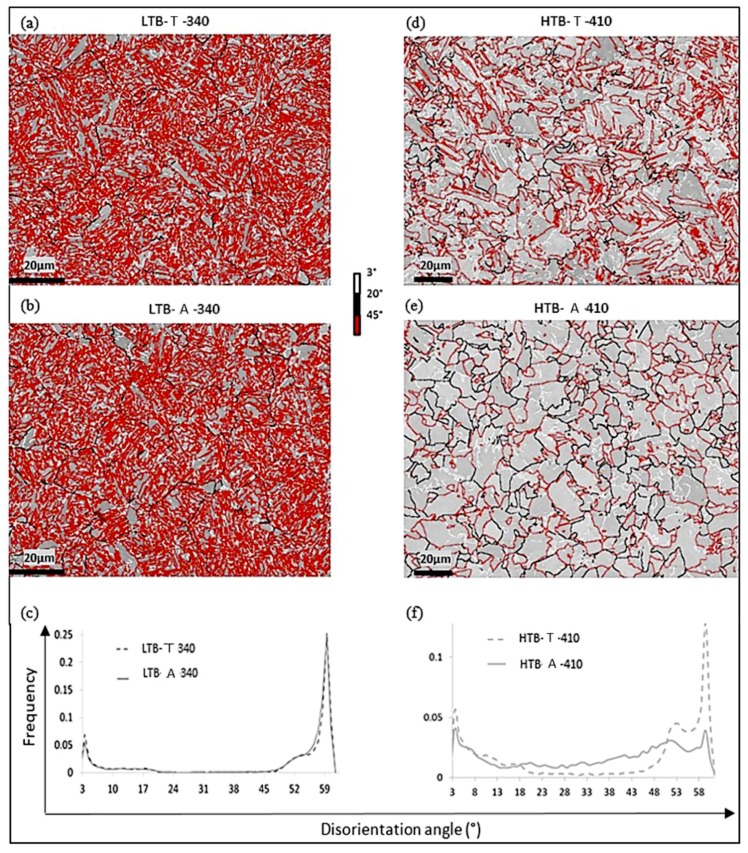
Angular disorientation maps (with the associated color code shown on the figure), comparing LTB-T (**a**) with LTB-A-340 (**b**) and HTB-T (**d**) with HTB-A-410 (**e**), (**c**) and(**f**) angular disorientation histograms associated with the EBSD data.

Figure 3 compares the EBSD results for a transformed (T) and an aged (A) microstructure respectively for LTB (a,b,c) and HTB (d,e,f) microstructures. For this first comparison, the ageing temperatures correspond to the transformation temperatures (340 °C and 410 °C respectively for LTB and HTB).

The LTB-T sample exhibited a lath microstructure with predominance of block boundaries (high disoriented boundaries, mainly of 60°, in red on the EBSD map). The coupled MERENGUE2/DECRYPT analysis indicated a 100% Type B variant organization which is typical of a lower bainite. Inter- and intra-lath carbides, characteristic of lower bainite according to Mehl [5] were also observed. The extended isothermal ageing (5 h at 340°C—H_HJ_ = 12.7) induced no substantial change in the ferrite matrix. Figure 3b shows that the LTB-A-340 microstructure is still homogeneous and presents a very pronounced lath structure. The disorientation distributions before and after ageing presented in Figure 3c are remarkably similar and confirm that the spatial organization of variants was not affected by the prolonged thermal treatment at 340 °C. Nevertheless, a meaningful decrease in hardness between LTB-T and LTB-A-340 was observed (from 347 HV to 326 HV). It can be attributed to the dislocation recovery in the ferrite matrix.

In contrast, the HTB microstructures exhibited a decomposition of the ferrite matrix into a granular structure only after 950 s at 410 °C (H_HJ_ = 13.3). The coupled MERENGUE2/DECRYPT analysis indicated that the HTB-T microstructure was a mixture of Type A and B. The respective proportion of Type A and B was 70% and 30% in the transformed state. Moreover, domains containing only inter-lath carbides (characteristic of upper bainite according to Mehl classification) and domains containing both inter and intra-lath carbides (characteristic of lower bainite according to Mehl classification) were observed in the microstructure. It can be thus concluded that the as-transformed HTB-T microstructure is a mixture of upper and lower bainite.

**Figure 4:**
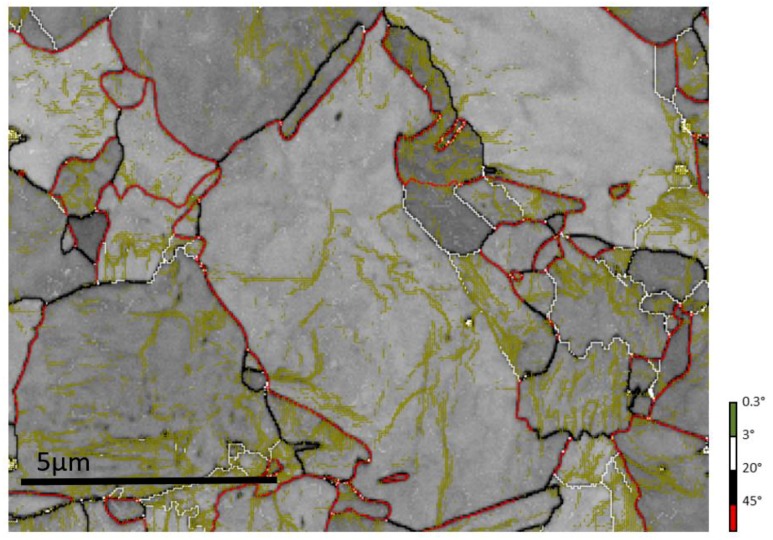
Enlarged area taken from the EBSD map of the HTB-A-410 highlighting in green very low disoriented sub-boundaries (between 0.3° and 3°) present in the granular structure. The lath structure has completely disappeared after this granularization process.

After 950 s of ageing (H_HJ_ = 13.3), the HTB-A-410 microstructure was significantly different from the HTB-T one: the acicular blocks (essentially those disoriented by 60°, in red in Figure 3d) disappeared in favor of a granular structure (see Figure 3e). This evolution is also detected in the disorientation histogram of Figure 3f, by the significant decrease in the frequencies of 60° disorientations (against an increase in the relative frequencies of random disorientations between 20° and 45°). This ferrite decomposition resulted in a granular structure containing a network of low disoriented sub-boundaries. Since very low disorientations down to 0.3° were detectable, such sub-boundaries were visible on the EBSD map as shown in Figure 4. The final mean size of the granular structure was close to the packet size of the as-transformed microstructure. It must be emphasized here that the entire microstructure, was affected by this ageing evolution, even Type B domains. This microstructure evolution produced a drop in hardness of 60HV, which is far higher than in the case of the sole recovery at 340 °C. The same phenomenon was already observed in Fe-Ni-C steel with lower carbon content [28].

### 3.2. Evolution of Martensite-Type Microstructures during Ageing

**Figure 5:**
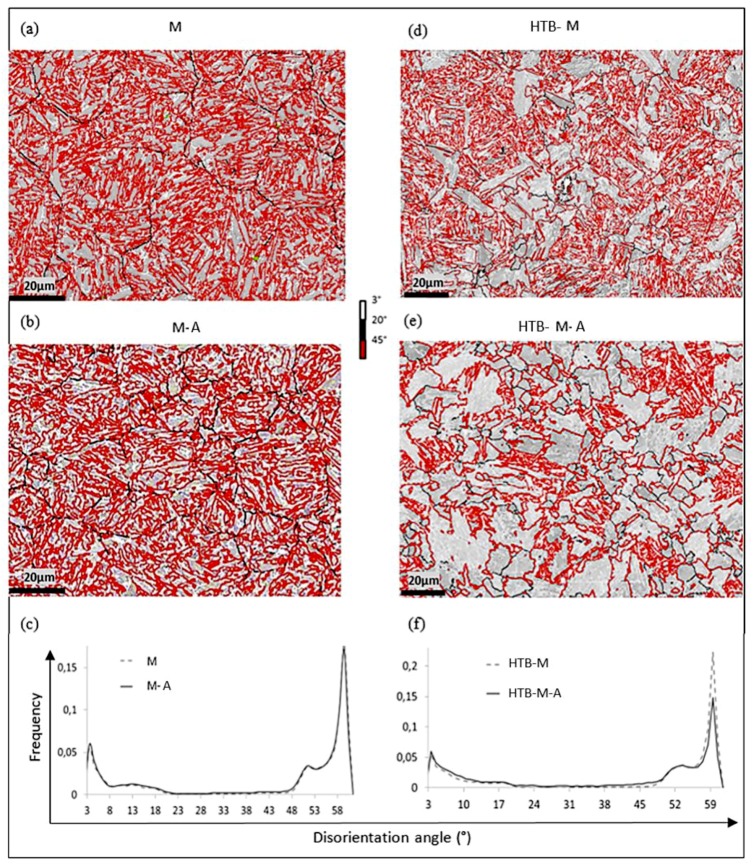
Angular disorientation maps (with the associated color code shown on the figure), comparing: M (**a**) with M-A (**b**); and HTB-M (**d**) with HTB-M-A (**e**). (**c**,**f**) Angular disorientation histograms associated with EBSD data.

The martensite microstructure M obtained by direct quenching (shown in Figure 5a) had a crystallographic variant arrangement of Type B, in accordance with the prior works of Morito et al. [16]. After 5 h ageing at 340 °C, it remained unaffected, as seen with the microtextures of the M-A sample in Figure 5b and the corresponding disorientation histograms in Figure 5c.

As expected, the as-transformed HTB-M microstructure was made of both coarse domains containing low disorientated boundaries (formed presumably at 410 °C) and very fine lath structures (formed during quenching) (as seen in Figure 5d). The coupled MERENGUE2/DECRYPT analysis confirmed that the structure was composed of 42% Type A and 58% Type B. This ratio is consistent with the considered heat treatment. As a rule of thumb, HTB-M microstructure was in fact expected to contain 60% of high temperature bainite (HTB-T)—made, respectively, of 70% Type A and 30% Type B—and 40% of martensite (100% Type B).

After ageing at 340 °C for 5 h, the HTB-M-A microstructure exhibited a remarkable decrease in the number of block boundaries compared to HTB-M, as seen in Figure 5d,e. Again, it is mostly due to the decrease in frequency of 60° boundaries, as evidenced by the superposed disorientation histograms in Figure 5f.

### 3.3. Influence of the Ageing Temperature on the Stability of As-Transformed Bainite Microstructures

At this step, it cannot be excluded that the microstructure evolution observed for HTB-A-410 and HTB-M-A could be triggered by the ageing temperature thanks to a thermally activated mechanism regardless of the microstructure. Consequently, additional treatments were performed by varying the holding temperatures once the transformation was finished (see Table 1a,b).

**Figure 6:**
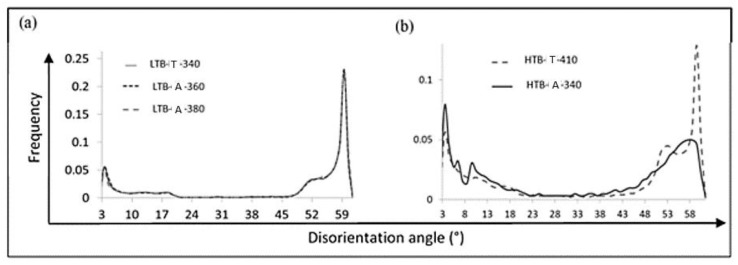
Angular disorientation histograms of: (**a**) LTB-T, LTB-A-360 and LTB-A-380; and (**b**) HTB-T and HTB-A-340 samples.

First, the LTB-T microstructure was aged at higher temperatures than 340 °C. Figure 6a shows the disorientation histograms obtained on the LTB-A-360 and LTB-A-380 samples which were isothermally held after transformation, at 360 °C for 5 h and at 380 °C for 30 min, respectively. In both cases, the H_HJ_ parameters of 12.9 and 13.1 were close to the ones applied to HTB-A-410 (H_HJ_ = 13.5) and HTB-M-A (H_HJ_ = 12.7) for which granularization was observed. Whatever the applied heat treatments, the disorientation histograms are strictly similar. It means that, in both cases, the ferrite matrix does not evolve despite a severe treatment. The corresponding hardness decrease after ageing at 360 °C was similar to the one observed after ageing at 340 °C. The mechanisms behind this decrease were probably similar, i.e., the recovery of the ferrite matrix.

Secondly, the isothermal ageing of the HTB-T sample was performed at a lower temperature. The bainite microstructure fully transformed at 410 °C after 80 s was held at 340 °C for 870 s (sample HTB-A-340). Figure 6b compares the disorientation histogram obtained for both samples. One can notice a remarkable decrease in the 60° peak. This result highlights the fact that, in presence of Type A microstructure, the lath decomposition starts at this low temperature (340 °C, i.e., Ms + 15 °C) and after a short equivalent time (H_HJ_ = 11.9 only).

## 4. Discussion

### 4.1. Key Role of the Initial Microstructure Regardless of the Ageing Temperature

This paper demonstrates that, in Fe-5Ni-0.33C (wt %) steel, lower bainite (Type B microstructure) is stable during isothermal holdings at both low and high temperatures. Yet, some microstructure evolutions are still possible at atomistic or nm-scale since the microstructural transformations presented were performed by SEM. However, lower bainite (Type B microstructure) evolves as soon as it contains a fraction of upper bainite (Type A microstructure). This evolution consists mainly in the decomposition of the block structures (disappearance of 60° disoriented boundaries) in favor of a polygonal structure containing a network of sub-boundaries. This is the reason we have qualified this process as “granularization”. Martensite behaves similarly as lower bainite under low temperature tempering—its ferrite matrix is stable—while, in the presence of upper bainite, it decomposes under the same tempering conditions as lower bainite. This decomposition is not limited to the fraction of Type A bainite; the entire microstructure is affected by the process.

This result suggests that the as-transformed microstructure plays a key role to trigger the granularization process. Temperature is thus not the sole decisive parameter promoting the evolution. For the studied steel, as well as the previous one with slightly lower carbon content [28], the decrease in mechanical properties can be observed for H_HJ_ tempering parameters in the range of 11.8–13.2, far smaller than the value of 18 expected by the seminal work of Hollomon and Jaffe [25]. Consequently, their approach is questionable for two aspects:

(1) Critical tempering conditions that degrade the steel mechanical properties are not valid for all steel compositions, particularly for FeNiC alloys.
(2) Equivalent tempering parameters without considering the microstructure type seems not relevant.

### 4.2. Effect of the Steel Composition

In the studied FeNiC systems (Fe-0.13C-5Ni previously [28] and Fe-0.33C-5Ni in this paper), the carbon content seems to have an impact only on the phase transformation kinetic. It is a known effect of carbon: the higher the carbon content, the slower the bainite transformation [20]. For our studies, fully lower bainite was obtained after only 50 s in the 0.13 C steel, whereas 1700 s were necessary for the 0.33 C steel. However, the granularization process seems not highly sensitive to the carbon content. Indeed, for both steels, the microstructure became granular for ageing conditions with similar low Hollomon—Jaffe parameters (between 12.5 and 13).

On the contrary, substitutional elements seem to have a remarkable effect on the granularization kinetics. In fact, Mn based alloys studied in the literature are reported to undergo a granularization only after far longer ageing and at higher temperatures, i.e., in more severe conditions (typically for H_HJ_ higher than 16–18). It is thus probable that Mn is an alloying element reducing the kinetics of granularization contrarily to Ni. This difference in the behavior between both substitutional elements is not surprising as they do not affect the mobility of interfaces in the same way during ferrite transformation and conventional recrystallization [31,32]. Consequently, it is likely that the mobility of high angle boundaries in the granularization process is controlled by a solute drag mechanism, as in a more conventional recrystallization process and despite the low temperature [33,34,35]. Uhm et al. [35] showed that Mn and Ni affect differently the activation energy of α/α interface mobility. Compared to Ni, Mn slows further this mobility down. We predict that granularization can occur in Mn-containing alloys, but with probably slower kinetics than in Mn-free alloys.

### 4.3. Granularization and Bainite Classifications

According to the literature, granular bainite formation has only been observed during continuous cooling treatments. In fact, Bhadeshia reported that granular bainite occurs only in continuously cooled low-carbon steels and explained that it cannot be produced by isothermal transformations [19]. A similar conclusion was drawn by Krauss et al. [36].

Interestingly, we obtained granular bainite from the granularization of lath-like structures by isothermal treatment. The final microstructure is described as equiaxed ferrite grains having the average size of prior packets, presenting low disoriented sub-grain boundaries and containing inter- and intra-granular carbides (cementite). The isothermally aged and granularized microstructures thus show many similarities with granular bainite.

In our opinion, it suggests that the formation of granular bainite can also be explained by the granularization of an intermediate lath structure (mainly of Type A, of course). This assumption opens new roads for discussing granular bainite formation mechanisms.

## 5. Conclusions

It has been proven that, for Fe-0.33C-5Ni steel, mixed upper and lower bainite microstructures show a remarkable evolution when aged. They evolve quickly toward a granular structure. This evolution is also observed in model microstructures made of upper bainite and fresh martensite.

On the contrary, lower bainite and martensite do not undergo any morphological or microtextural evolution when they are subjected to isothermal holding (even at temperatures above their transformation temperatures), at the studied micrometer scale.

This granularization process on the ferrite matrix consists in the disappearance of all acicular block boundaries (high disoriented) and lath boundaries (low disoriented). This process is triggered by the presence of upper bainite and affects the entire microstructure, even that made of lower bainite or martensite.

This phenomenon is accompanied by a significant drop in hardness, higher than the sole recovery effect.

The process seems to be controlled by both boundary energies and sizes of the involved features (packets, blocks, etc.) and requires a deeper investigation. The results give new paths for discussing the formation of granular bainite.

## Figures and Tables

**Table 1:** Main parameters of the heat treatments to obtain: (**a**) HTB-type microstructures; (**b**) LTB-type microstructures; and (**c**) martensite (M)-based microstructures (T stands for as-transformed and A for as-aged conditions).

	Microstructure		Heat Treatment Temperature/Time	State	Hardness (HV)	H_HJ_
(a)	High Temperature Bainite	HTB-T	410 °C/80 s	Transformed	302	12.5
		HTB-A-410	410 °C/80 s + 410 °C/870 s	Aged	242	13.3
		HTB-A-340	410 °C/80 s + 340 °C/870 s	Aged	264	11.9
(b)	Low Temperature Bainite	LTB-T	340 °C/1700 s	Transformed	347	12.0
		LTB-A-340	340 °C/80 s + 340 °C/5 h	Aged	326	12.7
		LTB-A-360	340 °C/80 s + 360 °C/5 h	Aged	315	13.1
		LTB-A-380	340 °C/80 s + 380 °C/1800 s	Aged	320	12.9
(c)	Martensite based microstructure	M	Quenching	Transformed	520	0
		M-A	Quenching + 340 °C/5 h	Aged	460	12.7
		HTB-M	410 °C/25 s + Quenching	Transformed	400	-
		HTB-M-A	410 °C /25 s + Quenching + 340 °C/5 h	Aged	352	12.7
